# Inhibition of Particle Growth During Single‐Pulse Laser Fragmentation by Barrierless Adsorption of the Just‐Formed Gold Nanoparticles on Graphene Oxide

**DOI:** 10.1002/cphc.202500671

**Published:** 2026-03-03

**Authors:** Meike Tack, Anton Plech, Yogesh Pokhrel, Maron Dolling, Martin Ahrens, Gereon Hüttmann, Sven Reichenberger

**Affiliations:** ^1^ Technical Chemistry 1 University of Duisburg‐Essen Essen Germany; ^2^ Institute for Photon Science and Synchrotron Radiation Karlsruhe Institute of Technology Eggenstein‐Leopoldshafen Germany; ^3^ Institute for Biomedical Optics University of Lübeck Lübeck Germany; ^4^ Medical Laser Center Lübeck GmbH Lübeck Germany; ^5^ German Center for Lung Research (DZL) Grosshansdorf Germany

**Keywords:** coalescence, colloids, immobilization, metal clusters, size quenching

## Abstract

The use of pulsed lasers to produce surfactant‐free colloidal nanoparticles via laser ablation and fragmentation is well established. For laser ablation, the addition of support materials during laser ablation in liquids has shown size quenching effects, directly yielding supported and surfactant‐free ~7 nm metal nanoparticle catalysts in a single step. However, its feasibility for laser fragmentation has barely investigated. This is why, in this work, we show that the fragmentation of initial, large gold nanoparticles (AuNPs), supported on graphene oxide (GO) already before laser fragmentation, leads to a significant size quenching effect, yielding GO‐supported sub 3 nm gold clusters. The size‐quenching was found to be particularly effective when the mass load of gold on GO was below 10 wt%, and a diluted Au/GO dispersion was used. In this context, the role of GO sheet‐to‐sheet distance and the overall gold concentration are discussed and used to predict experimental conditions which lead to a minimal AuNP size. The presented study therefore, not only optimizes the synthesis conditions to gain GO‐supported clusters but also presents a new study concept to mechanistically investigate the post‐synthesis growth processes during surfactant‐free laser‐based synthesis of nanoparticles.

## Graphical abstract

Supporting the initial gold nanoparticles onto graphene oxide sheets before laser fragmentation with low spatial density leads to a significant reduction in the final particle size, especially at low mass loads of gold (higher interparticular distance).

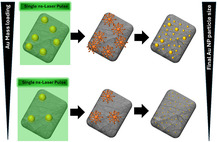

## 1 Introduction

Laser ablation and laser fragmentation in liquids (LAL/LFL) are well‐established methods to produce pure, surfactant‐free, and catalytically active nanoparticles [1, 2]. To increase the content of the especially active size fraction below 10 nm in the gained colloid [3] or to even obtain fully inorganic but fluorescent nanoclusters [4], the inhibition of particle growth has to occur already during the laser synthesis [5]. For tailoring this inhibition of cluster growth, it is important to look at the time scale on which cluster growth occurs. Advanced time‐resolved small‐angle scattering (SAXS) studies can elucidate the cluster growth directly after the laser irradiation, allowing to determine the time scale after which the growth inhibition takes effect. For the LAL of a gold target, Letzel et al. could show that the growth inhibition of clusters with sodium chloride already happens in the cavitation bubble [6]. For LFL of gold nanoparticles (AuNPs), Ziefuss et al. could observe nanoparticle growth in a time frame of 30 ns up to 10 µs delay for nanoparticles fragmented in pure water, whereby the results have shown indications that a stopped growth of the clusters occurred at a delay of ~1 µs when 300 µM NaCl/NaOH was present as growth quenching agent during the laser fragmentation [7]. Theoretical models of the laser fragmentation of nanoparticles predict that the presence and growth of clusters already occur on the picosecond time range, as shown for the laser fragmentation of a modeled 20 nm AuNP by Zhigilei et al. [8, 9]. For measurements on a longer (>seconds) time range, UV–vis measurements allow the simple observation of nanoparticle size. As an example, platinum nanoparticles were observed to grow for even hours and days after the laser fragmentation [10]. To inhibit such particle growth processes after laser fragmentation of surfactant‐free, electrostatically stabilized colloids without the addition of organic surfactants, two strategies come to mind: (1) improve the electrostatic repulsion between the formed clusters or (2) immediately immobilize the clusters on a support material already during laser fragmentation.

For strategy (1), several growth inhibitors were already described in the literature. Mostly, micromolar amounts of electrolytes, which induce and enhance electrostatic repulsion, are used. Rehbock et al. investigated the addition of several mono‐ and multivalent salts with different concentrations during the laser ablation of a gold target. As a result, monovalent NaCl and NaBr in particular showed excellent particle stabilization properties, even at low micromolar concentrations, leading to smaller particles compared to using pure water [11]. Sylvestre et al. observed the same trend and explained the size quenching with an increased electrostatic repulsion of the already partially oxidized AuNPs [12]. This repulsion can also be enhanced when altering the pH‐value of the colloid, as this (de)protonates the particles´ surface groups [12, 13]. Consequently, Ziefuss et al. observed particularly small particle sizes (<3 nm) and high mass yields of laser‐generated gold clusters (>70%) when the LFL was conducted in a mixture of 300 µM NaCl and 300 µM NaOH, which was discussed to drastically increase the AuNPs’ Zeta potential and hence electrostatic repulsion [5]. Of course, if the presence of organic surfactants is unproblematic or even desired, a steric (as well as electrosteric) stabilization strategy can be employed via ligand addition. Especially for biomedical applications, the addition of biomolecules to the ablation (or fragmentation) liquid enables a one‐step synthesis of producing and modifying the nanoparticles, while simultaneously achieving a smaller particle size [14, 15]. Besides biomolecules, long‐chain organic molecules like sodium dodecyl sulfate (SDS) can be used for size‐quenching [16, 17].

Strategy (2), namely the deposition on supports already during laser fragmentation, may represent another valuable option to stabilize the small nanoparticle fragments and directly obtain, e.g. a supported nanoparticle catalyst at the same time. Past studies on the laser ablation of metals in liquids have used colloidal dispersions of supports like (reduced) graphene oxide or silica [18, 19, 20, 21, 22] during laser synthesis because the as‐produced clusters directly adsorb on the support. This results in smaller particles as coalescence is stopped by immobilization. Additionally, using carbon‐based support materials such as graphite or graphene as size quenchers has several advantages for potential applications, like chemical robustness in acidic and alkaline media, low price, high surface area, and high conductivity [23, 24]. By introducing oxygen groups into the graphene structure, graphene oxide can be formed [25]. This oxidized form is hydrophilic, resulting in good dispersity in water. Haxhiaj et al. showed that the amount of reduced graphene oxide (rGO) used as a support material during the laser ablation of platinum influences the gained nanoparticle size, with a decreased particle size when increasing the amount of support material [21]. Yet, so far, such size quenching effects have not been reported or studied in the context of laser fragmentation in liquids. In a first attempt, we recently published a study where colloidal ~60 nm AuNPs and GO sheets were simply mixed and then laser‐fragmented. The evolving cluster size was observed via insitu SAXS measurements, and a comparison to the cluster size achieved during the fragmentation of pure ~60 nm AuNPs showed larger particles in the sample containing GO. Therefore, we hypothesized that the unexpected accelerated particle growth is due to an electrostatic confinement, given that the AuNPs and GO sheets both obtained a Zeta potential <−30 mV [26].

Building on those previous results, we adopt a distinct approach in this study. Unlike our previous SAXS/WAXS study, which utilized a simple colloidal mixture of negatively charged AuNPs and negatively charged GO [26], we employed our recently developed barrierless self‐assembly method [27] to pre‐adsorb ~60 nm AuNPs onto thin GO sheets, despite their electrostatic repulsion, prior to laser fragmentation. This ensures spatial proximity between GO sheets and the resulting Au clusters. Additionally, to further facilitate Au cluster adsorption onto GO sheets, we conducted the fragmentation at a high ionic strength (200 mM NaCl), which is the same ionic strength as we used to support the initial AuNPs on GO. Our hypothesis is that by circumventing the electrostatic repulsion, the clusters directly adsorb onto the GO sheets in the same barrier‐less manner as during the pre‐adsorption of the ~60 nm AuNPs. By varying the initial AuNP mass loading on GO, we investigated the effects of Au cluster density (dependent on AuNP concentration and spatial closeness) and GO sheet distance (dependent on GO concentration) on the final particle size. This allowed us to determine which Au/GO mass loadings and concentrations are needed to gain a minimal particle size.

## 2 Experimentals

### 2.1 Synthesis and Support of the Initial AuNPs onto the GO Sheets

AuNPs were synthesized by laser ablation in liquid (LAL) of a gold target immersed in an aqueous, 0.1 mM sodium chloride solution using a Nd:YAG laser (Ekspla, model: Atlantic) at 1064 nm. The obtained gold colloid was further processed with a dedicated centrifugation protocol to extract the ~60 nm AuNP size fraction. To remove the smaller AuNPs below 40 nm, the particles were centrifuged, starting with one centrifugation in 50 mL Falcon tubes at 377G for 80 min (Hettich Universal 320 centrifuge). The residue was then centrifugated two times for 30 min at 358 G in 1.5 mL Eppendorf reaction vessels (Hermle Z 216 MK centrifuge). Between each centrifugation, the supernatant (containing the small AuNP) was discarded, while the larger, sedimented AuNP were redistributed in a solution containing 0.1 mM NaCl (pH value ~6.5) via shaking and subsequent treatment in an ultrasonic bath to separate possible agglomerates. During the second part of the centrifugation protocol, the larger AuNPs >100 nm were removed via a 5 min centrifugation at 377 g in 50 mL Falcon tubes (Hettich Universal 320 centrifuge), whereby the desired ~60 nm size fraction was now obtained from the supernatant after the single centrifugation step. The 60 nm AuNP were then used as a defined model for the fragmentation experiments (after barrierless self‐assembly onto the GO sheets [27]).

For the graphene oxide sheets, a GO paste (Sigma–Aldrich, Art. No.: 900 704) was dispersed in MilliQ water (18.2 MΩ) with a concentration of 1 g/L. Exfoliation was achieved via stirring and subsequent ultrasonification (Bandelin, Sonopuls 2070, 3 burst pulse mode at 20 W) in water in continuous mode for 2 h with appropriate cooling periods of 5 min (every 10 min of sonication) to avoid heating up the dispersion. Supporting the initial AuNPs onto the GO surface was conducted via electrostatic charge screening in accordance with an earlier demonstration [27]. In short, the dispersion of exfoliated GO sheets (33 mg/L after AuNP addition) was first mixed with 4 M sodium chloride (200 mM concentration in the finished composite). Then, the 60 nm AuNPs were added dropwise (~1 ml/min) into the GO dispersion (under vigorous stirring), until the desired mass loading was reached (2–35 wt% → calculated via (mass_Au_)/(mass_Au_+mass_GO_); see Table S1 and S2 for more detailed information). This range of mass loading was chosen to produce samples with different surface loadings as well as different spatial distances between the adsorbed AuNPs, which will change the produced cluster density during fragmentation. In particular, the upper limit for the mass load (of 35 wt%) was selected, given that our previous study has hinted at an increasing particle agglomeration at a mass load of 40 wt%. The lower limit was, in turn, selected to ensure average interparticle Au–Au particle distances that are as large as possible, while a sufficient amount of particles was still visible during transmission electron microscopy (TEM) analysis. Due to the high ionic strength in the GO dispersion, the AuNPs become unstable immediately after being immersed in the dispersion. This instability forces the AuNPs to either agglomerate or adsorb onto the support material. To minimize AuNP agglomeration, the AuNPs were added slowly (~1 ml/min) to keep the overall concentration of free (non‐Tadsorbed) AuNPs in the AuNP–GO mixture as low as possible. This way, the adsorption of the AuNP on the GO support material is expected to be statistically favored (see Ref. [27]). One day after the adsorption, the Au/GO particles were centrifuged to concentrate them while maintaining the 200 mM NaCl concentration. Because of the large surface area of the GO sheets, not all Au/GO particles were sedimented to the bottom of the centrifugation tube, which led to a limited loss of particles. Higher centrifugal forces (>1500 G) would have enhanced aggregation of the loaded GO sheets. Therefore, the samples were examined through UV–vis spectroscopy (Thermo Scientific Tabletop device) to determine the concentration of the AuNPs (see Figure S1 and Table S2) after centrifugation.

### 2.2 Laser Fragmentation in Liquids (LFL)

The pulsed laser fragmentation of the supported AuNPs was conducted in a flat jet, which ensures a uniform laser fluence within the irradiated dispersion [28]. A Nd:YAG laser (Edgewave) at 532 nm was used at a fluence of 1.3 J/cm^2^, a repetition rate of 50 kHz, and a pulse duration of 10 ps. The laser beam was shaped by two cylindrical lenses to define the required laser fluence. The Au/GO particle suspension was pumped through a flat‐jet nozzle (company: BETE, thickness ~30 µm, see section S2 in the SI for the thickness measurement by optical coherence tomography) by an air pressure‐based pump system. The volume flow rate of the AuNP and the repetition rate of the laser were adjusted to ensure that each laser pulse irradiated a new volume element, ensuring single laser pulse processing conditions (similar to previous studies [28, 29, 30]). In the first part of this study, the fragmentation of Au/GO composites with different gold mass loadings, but similar AuNP‐concentration (~3 mg/L) and containing 200 mM NaCl is investigated. In the second part of this study, Au/GO with the same mass loading of gold (25 wt%) but different overall Au/GO mass concentrations (and hence also different gold concentration) were fragmentated (AuNP‐concentration: 0.3–5.8 mg/L), again containing 200 mM NaCl. With this experiment, the influence of different GO sheet distances (which changes when the GO concentration is altered) on the gained gold cluster size is analyzed. The high salinity (200 mM NaCl) hereby ensures that the Au clusters, generated during LFL, are forced to adsorb back onto the GO sheets. The optimal ionic strength of 200 mM NaCl was identified in an earlier study [27]. To show that the GO sheets act as size quenchers, we also fragmented the AuNPs in pure water with the same laser parameters but 10 mg/L gold concentration as a reference case.

### 2.3 Particle Size Determination

The Au/GO samples were characterized by HR‐TEM analysis (JEOL 2200JS, C_s_‐aberration corrected TEM, 200 kV acceleration voltage) after the supporting and centrifugation, and after the laser fragmentation. For the sample preparation, Ni Lacey Carbon Grids (Plano GmbH) were put on filter paper before several tens of microliters were dropped onto the grid. This method minimizes drying‐induced agglomeration, as excess liquid is directly removed. By washing the grids 10–20 times, the remaining sodium chloride was removed. The size histograms (see supporting information) were fitted by a log‐normal distribution.

### 2.4 Time‐Resolved SAXS for Fragment Size Determination

The Au/GO nanoparticle suspension (25 wt% gold mass loading with 300 mg/l GO, particles suspended in MilliQ water or 200 mM NaCl) was pumped through a circular liquid jet (thickness 270 µm) to replace the liquid between two laser shots. The irradiation was achieved by exposing the liquid with 1 ps laser pulses (400 nm, 1 kHz, Coherent regenerative amplifier) at a fluence of 0.16 J/cm^2^, which is sufficient for full fragmentation [31]. Time‐synchronized X‐ray pulses at the European Synchrotron (beamline ID09) of 100 ps duration scatter from the laser‐irradiated volume and are detected by an X‐ray 2D detector (Rayonix). The scattering signal from 0.17 to 0.5 Å^−1^ was integrated azimuthally and analyzed by a SAXS function with a model‐free assumption of a particle sizes [31]. The experiment allows deriving the average diameter of the created clusters with high time resolution. Here, we used a delay of 3–20 µs after excitation. Error bars were derived from repeated experiments. More details can be found at [7, 31]. A comparison of the fit with a reverse Monte Carlo (rMC) approach of fitting the measured data by an arbitrarily varied size distribution of spherical clusters [32] has been undertaken.

## 3 Results and Discussion

**FIGURE 1:**
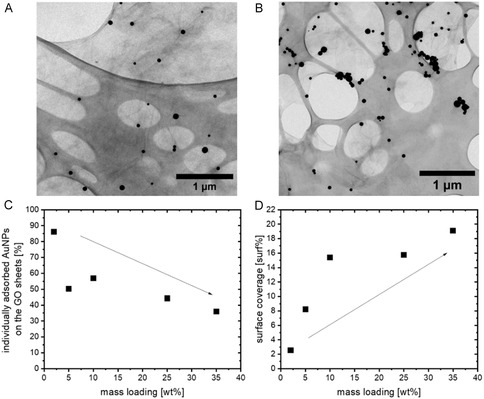
Exemplary TEM pictures of ~60 nm Au on GO sheets with (A) 2 wt% and (B) 35 wt%, (C) percentage of single‐adsorbed AuNPs in dependence of the mass loading, and (D) surface coverage of all adsorbed AuNPs on four 2 × 2 µm^2^ GO sheet areas in dependence of the mass loading. The large‐scale oval structures stem from the lacey carbon grid, while the filamentous tint between the NPs shows the GO sheets.

In line with our previous study, the laser‐generated, colloidal ~60 nm AuNPs that were obtained from the centrifugation protocol were supported onto the graphene oxide sheets via a barrierless self‐assembly method [27] with several mass loadings ranging from 2 to 35 wt%. The obtained Au/GO hybrids were characterized by TEM analysis to count the number of individual AuNPs and the number of AuNPs that are in contact with other ones (agglomerates). For each sample, a sum of at least 250 nanoparticles was classified in this way. For the classification, the nanoparticles were categorized as either individual/isolated nanoparticles or nanoparticles in an agglomerate consisting of 2, 3, 4…, etc. nanoparticles. Additionally, we analyzed the data in two different ways: (1) with respect to the number of agglomerates in each size class, and (2) the overall number of nanoparticles in each size class. Counting the number of agglomerates or the number of nanoparticles may seem like a small detail, yet this distinction is important to evaluate both the number of agglomerates and the total cluster mass contained in these agglomerates. Please note that this analysis can potentially be affected by projection artifacts (i.e., false clusters) caused by GO sheet stacking during drying for the preparation of the TEM grid. To minimize such artifacts, we intentionally dropped a diluted Au/GO dispersion (<5 mg/L) onto the TEM grid that was located on a sheet of filter paper. The filter paper was used to directly remove most of the dispersion and keep the amount of adsorbed Au/GO on the TEM grid as low as possible. Figure 1A,B show a representative TEM image of the 2 and 35 wt% Au/GO hybrids, respectively, and used for this analysis. The TEM images of the other mass loadings (5, 10, and 25 wt%) can be found in Figure S3. From the determined number of nanoparticles in each category (individual or in an agglomerate containing 2, 3, 4… nanoparticles), a number‐weighted relative frequency of individual nanoparticles and respective AuNP agglomerate clusters on the GO sheets was calculated for each mass loading. The number frequency distributions of the agglomerates or the nanoparticles within the agglomerates can be found in Figure S4A,B, respectively. As can be seen from the number frequency plots in Figure S4, for all samples (different AuNP mass loading) the percentage of agglomerates (Figure S4A) as well as the percentage of all nanoparticles (Figure S4B) that are within the respective agglomerates (of 2, 3, 4, etc. NPs) exponentially decreases with increasing size of the Au NP agglomerates on the GO sheets. As one would expect, given that larger agglomerates host more nanoparticles (and are hence less abundant), the exponential decrease with increasing AuNP agglomerate size is significantly more pronounced when normalizing the analysis to the frequency of agglomerates (Figure S4A) instead of the frequency of nanoparticles (Figure S4B). Due to this statistical effect (and to avoid related misinterpretations), we will only focus on the frequency distribution that is linked to the number of nanoparticles (Figure S4B) in the following. We determined the total percentage of isolated (individual) AuNPs (values at *x* = 1 in Figure S4B) that were found on the GO sheets and plotted them with respect to the nominal mass loading of AuNP on GO in Figure 1C. As can be seen, the percentage of single‐adsorbed AuNPs drops linearly (monotonically) from 86% for the 2 wt% sample to 36% for the 35 wt% sample (with an outlier at 5 wt%), respectively.

To determine whether the agglomeration also affects (decreases) the surface coverage of the adsorbed AuNPs, we also measured the footprint of the AuNPs within the TEM images (or, in other words, the area of GO that is covered with nanoparticles). For this purpose, we chose four exemplary 2 × 2 µm^2^ sized areas in the TEM pictures and determined the sum of the projection area of each AuNP within this area (for every Au/GO mass loading). In Figure 1D, one can see that the measured surface coverage of the AuNPs increases with increasing mass loading. However, after a linear increase for mass loadings between 2, 5, and 10 wt%, the surface coverage seems to saturate for 25 and 35 wt%, which may again be linked to an increasing agglomeration tendency with rising nominal mass loading. This is supported when comparing the number frequency histograms of AuNP inside the respective AuNP clusters (see Figure S4B), where one can observe that the percentage of nanoparticles in clusters containing 3 or more AuNP linearly increases with rising mass loading. Note that many of those agglomerates are still small enough to show a plasmon resonance (see Figure S1) and hence be fragmented by on‐resonant (532 nm) pulsed laser irradiation.

**FIGURE 2:**
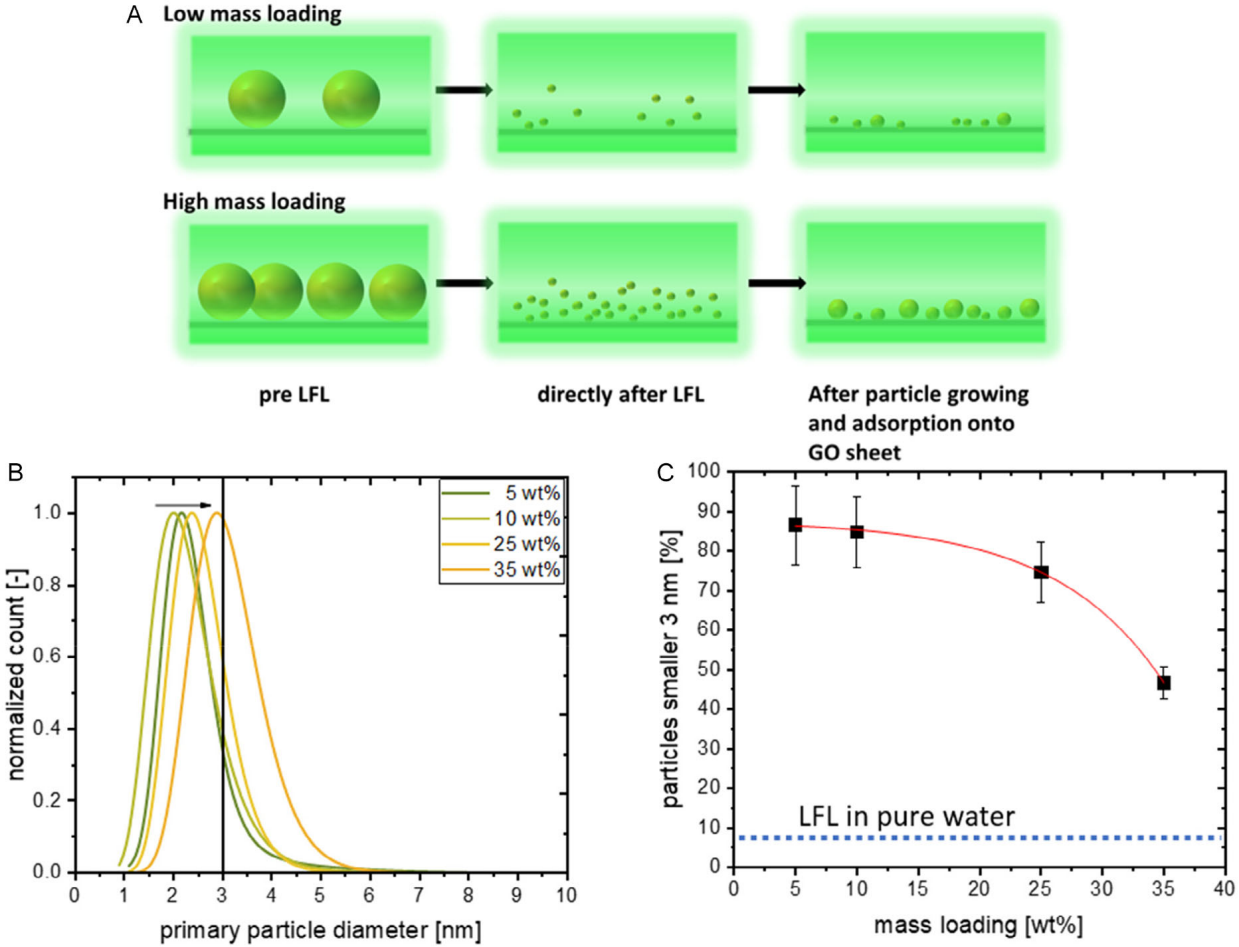
(A) LFL of Au/GO with ~3 mg/L initial AuNPs but different mass loading. Conceptional scheme of LFL of an Au/GO sample with a low mass loading and a high mass loading, (B) particle size distribution after LFL determined from TEM analysis (see Figure S6 for the corresponding histograms), (C) calculate percentage of generated AuNPs smaller 3 nm (the standard error results from the number of counted particles) with a comparison of the resulting particle size after AuNPs were fragmented in pure water (dashed line, but with a mass concentration of ~10 mg/L initial particles).

As stated in the introduction, we want to investigate two parameters. First, the effects of Au cluster density, which is dependent on the AuNP concentration and spatial closeness of the AuNPs immobilized on the GO sheets, and second, the GO sheet distance (dependent on the GO concentration) on the final particle size. To study those two parameters, we conducted two different laser experiments, where we used the same composites, which we had analyzed in Figure 1. First, we altered the mass loading of the Au/GO composites, but kept the mass concentration of the AuNPs constant (~3 mg/L, see Table S2 for exact values), and second, we kept the mass loading of the Au/GO composites constant but altered the overall Au/GO concentration during laser fragmentation. For the first experiment, as explained above, the spatial closeness and surface coverage of the initial AuNPs on the individual GO sheets increase with increasing mass loading. Therefore, we assume that the density of the generated clusters should increase for higher mass loadings. As an example, the number of, e.g. 2 nm clusters that can ideally be produced by fragmenting ~60 nm gold particles present on one GO sheet is 3.2 times higher for a mass load of 35 wt% compared to a 5 wt% Au/GO sample (see section S4 for the detailed analysis). Because of this higher number of clusters close to each other, the spatial distance between the gold nanoclusters formed via LFL would decrease. We assume that this promotes coalescence and growth processes, and, in turn, leads to larger fragments after LFL (see Figure 2A for a conceptual drawing). In Figure 2B,C the particle size distributions of the obtained AuNP fragments and the percentage of particle number fraction below 3 nm are shown, respectively. In accordance with our assumption, a higher gold mass loading and the related, increasing spatial proximity of the initial AuNP led to larger AuNP after LFL of the Au/GO composite (Figure 2B). Consequently, a lower mass percentage of generated gold fragments that are smaller than 3 nm can be observed in Figure 2C. In particular, nearly 85% of the generated particles are smaller than 3 nm when the gold mass loading on GO was set to 5 wt%. When increasing the mass loading by a factor of 5 (to 25 wt%), this percentage drops only slightly (down to ~75%). However, when further increasing the mass load to 35 wt%, less than 50% of the gold fragments were found to be smaller than 3 nm. In comparison, when the LFL is performed with unsupported (no GO present), colloidal ~60 nm AuNP and the same laser parameters, only 9.5% of the fragments are smaller than 3 nm (see dashed line in Figure 2C), and more importantly, a high number of large aggregated particle patches have formed. Corresponding exemplary TEM images and size distributions of the obtained AuNP after LFL can be found in Figures S5 and S6, respectively.

Although the stabilizing effect of GO sheets on AuNP fragments has been demonstrated, the underlying mechanism remains unclear. Notably, our previous study on the LFL of AuNP + GO mixtures suggested a caging effect of unbound GO, resulting in larger gold fragments when GO was present during LFL compared to LFL in pure AuNP [26].

During the experiments discussed in the context of Figure 2, we kept the initial AuNP concentration constant (~3 mg/L). Consequently, when varying the mass loading, the GO sheet concentration changed significantly from 52.8 mg/L for 5 wt% to 8.4 mg/L for 35 wt%. Therefore, it is not entirely clear if the observed correlations in Figure 2 are solely linked to the effect of the rising mass load (namely, the decreasing distance of the initial AuNP on GO and the decreasing unoccupied area on the GO sheets in case of higher mass loadings) or whether the overall GO content in the dispersion would also affect the stabilization of the gold fragments. Therefore, we conducted a second experiment, where we only used the Au/GO composite with a 25 wt% mass loading and altered the overall concentration of the Au/GO composites (0.3–5.8 mg/L initial AuNPs/0.8–15.6 mg/L GO) during the laser fragmentation experiments. This way, we now examine if the concentration of the GO sheets and thus the relative distance of the GO sheets in the dispersion would have any effect on the resulting particle size.

**FIGURE 3:**
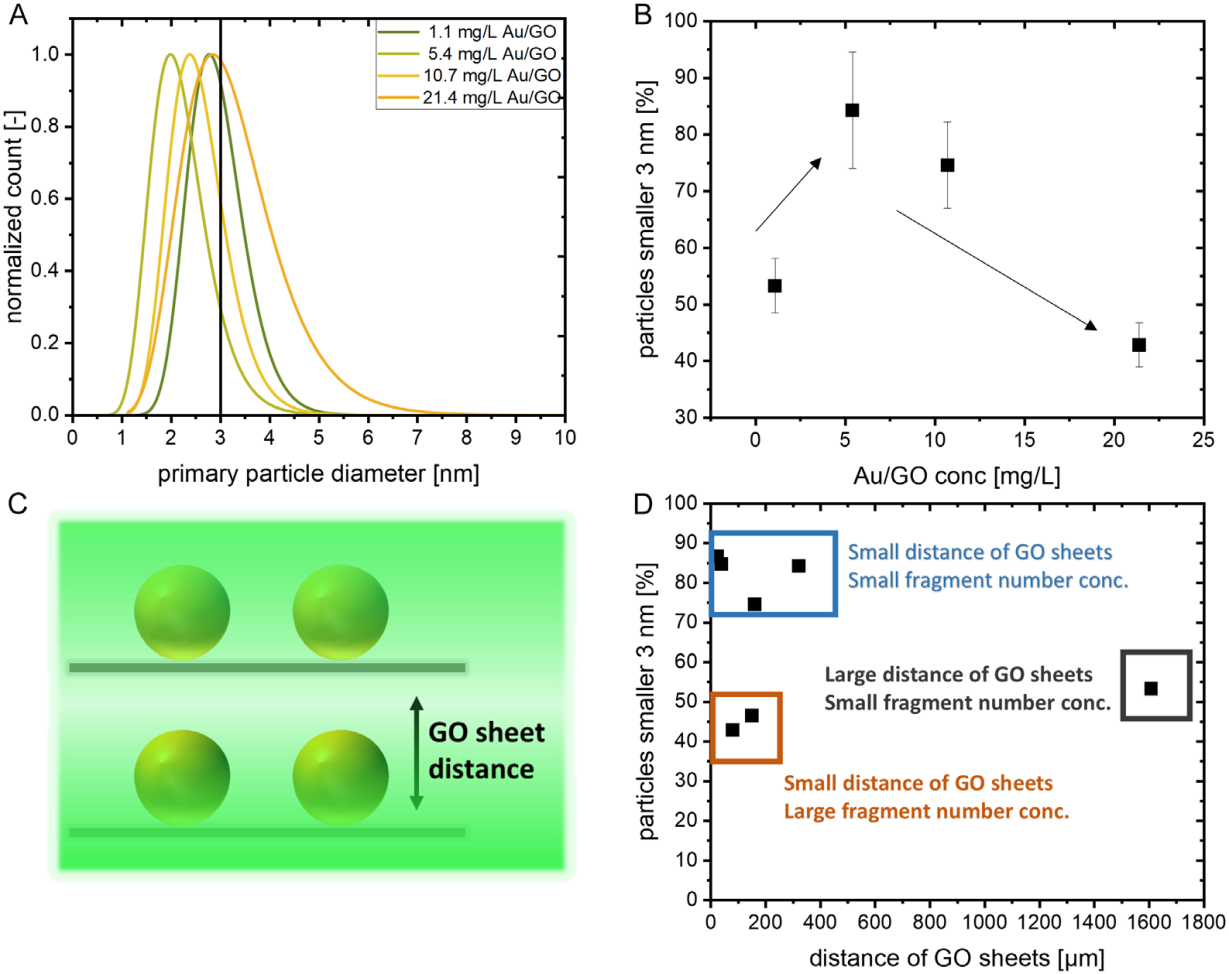
LFL of Au/GO with 25 wt% but different Au/GO concentrations. (A) Particle size distributions and (B) percentage of particles smaller 3 nm in dependance of the overall Au/GO (see Figure S7 and S8 for the corresponding TEM pictures and histograms, respectively), (C) scheme of the GO sheet distance (calculation in SI) and (D) summary of the percentage of the resulting particles smaller 3 nm (the standard error results from the number of counted particles) in dependance of the distance of the GO sheets in the corresponding samples.

Starting with the Au/GO sample concentration (10.7 mg/L) that was shown in Figure 2, we decided to not only investigate higher, but also lower Au/GO concentrations. The respective size distributions of the gold fragments and their number fraction on the GO sheets after LFL are shown in Figure 3A,B. As one can see, a minimal particle size (Figure 3A) and maximum fraction of gold fragments smaller 3 nm (Figure 3B) was reached when an Au/GO concentration of 5.4 mg/L_Au/GO_ (1.5 mg/L_Au_ and 3.9 mg/L_GO_) was selected. Higher or lower AuGO concentrations led to larger gold fragments and lower fractions of particles smaller than 3 nm.

To try and rationalize this observation, we assume two different, competing effects: For the smallest Au/GO concentration, the density of generated gold fragments is very small, which should hypothetically limit the coalescence and growth of AuNP fragments that were ejected into the liquid volume (see atomistic MD simulations from the Zhigilei group for particle growth simulations of single, unstabilized AuNPs [8, 9]). However, because of the low Au/GO concentration (1.1 mg/L), the related GO concentration is also very small (0.8 mg/L), and therefore, the average distance between the GO sheets in the liquids is large (see scheme in Figure 3C). We performed a simplified model calculation, which is described in section S6 of the SI. For this model calculation, we assumed an ideal exfoliation of the GO sheets and hence assumed monolayers and stacked GO sheets with equal distances. For the smallest Au/GO concentration of 1.1 mg/L (corresponding to 0.8 mg/L GO), the average distance of the GO sheets is ~1600 µm. We assume that the generated Au clusters not only adsorb on the GO sheet, from which they originate, but also on the surrounding sheets. Therefore, not only the generated cluster density, but also the distance between the individual sheets is crucial. With sheet‐to‐sheet distances of ~1600 µm, the Au fragments ejected by the LFL process either adsorb on the same sheet where they originate from or undergo enhanced ripening or even agglomeration before they can adsorb onto another sheet. When increasing the Au/GO composite concentration from ~1 to ~10 mg/L, the sheet‐to‐sheet distance decreases by one order of magnitude (from 1600 to ~100 µm, compare Figure S9. Consequently, at this concentration, a faster adsorption of the generated fragments and subsequent stabilization against post‐LFL growth processes (and hence smaller particle size) can be expected. But, as observed in Figure 3B, when reaching a composite concentration of 21.4 mg/L, the particle size increases again. Here, we assume the same coalescence effect that we described earlier. The spatial density of initial AuNPs on adjacent GO sheets directly affects the cluster concentration and therefore influences the cluster growth. Although we kept the ratio of Au to GO (and the number of gold fragments per GO surface) the same, the total amount of irradiated AuNPs is still increased due to the proximity of close sheets. Consequently, the likelihood for coalescence increases again if the overall Au/GO concentration becomes too high. We now combine the results from our two experiments (varying mass loading and varying overall Au/GO concentration) to identify the best conditions for the LFL and the reasons behind the observed growth quenching under certain Au/GO concentrations and gold mass loadings. For this, we first calculated the distance of the GO sheets in all our samples and plotted them against the produced cluster fraction below 3 nm (Figure 3D). We can classify the results into three different regions. First, two experiments showed a low number of particles below 3 nm, although the GO distance was small. In those experiments, either a high Au concentration or a high mass loading was used, leading to a high cluster density, which, in turn, promotes coalescence. Second, one experiment showed a large distance of the GO sheets, which allows the clusters to ripen before they attach to the next available sheet. Third, the best results were achieved using a GO concentration that leads to small distances between the sheets and an Au concentration/mass loading that results in a low cluster density (e.g. a mass loading of 5 or 10 wt% with an Au concentration of ~3 mg/L (Au/GO concentration 55.2 and 35.8 mg/L, respectively) or a mass loading of 25 wt% with an Au concentration of ~1.5 mg/L (Au/GO concentration 5.4 mg/L).

As we want to deepen the understanding of the diffusion processes of the clusters and GO sheets prior to an immobilization, we also estimated the speed of the clusters moving around in the liquid. Epple et al. used the advanced H‐NMR technique DOSY (diffusion ordered spectroscopy) to analyze the diffusion speed of ligand‐functionalized nanoparticles. As a result, for a 1.8 nm cluster, a diffusion coefficient of 2.3·10^−10^ m^2^/s was measured [33], from which a diffusion speed of around 15 µm for 1 s can be calculated. As the fluence used for the fragmentation leads to a phase explosion [31], directed movement of the clusters following the phase explosion has to be considered. Atomistic simulations of the LFL of a 20 nm AuNP have shown that the expelled gold material only travels ~50 nm (within the first ~14 ns) [8] and this travel does not exceed a distance of 120 nm during the ns time frame [9]. Because the sheet distances of the GO are in the micrometer range, those movements caused by the LFL process are not dominant, making the cluster diffusion the main reason for particle movement, and the GO sheet distance a crucial parameter, as for higher sheet distances (lower GO concentration), the diffusion time of the clusters to the next GO sheet increases. Interestingly, not only the two samples with GO sheet distances with a nanoparticle diffusion length as above (at ~24 and ~39 µm distance) show a high percentage of particles below 3 nm, but also the samples at 161 and 322 µm GO sheet‐to‐sheet distance led to 75 and more % of particles below 3 nm. Given the diffusion speed of small gold nanocluster determined via DOSY (~15 µm/s), and the assumption that the GO sheets are uniformly distributed in a sheet‐like configuration (leading to the aforementioned characteristic length scale), the expected diffusion‐ and hence stabilization time would be on the order of seconds. As we used a high ionic strength (200 mM NaCl) during the fragmentation, having a stabilization time of seconds could already lead to aggregation of the just‐formed clusters because of charge screening. However, the addition of a high saline concentration is crucial to induce quick re‐attachment of the fragments after they were detached from the GO sheets. We concluded this by performing the same Au/GO fragmentation as described above with 25 wt%, but instead of a 200 mM NaCl solution, we washed the Au/GO composite repeatedly with water via centrifugation and therefore performed the fragmentation in MilliQ water (Figure S10).

**FIGURE 4:**
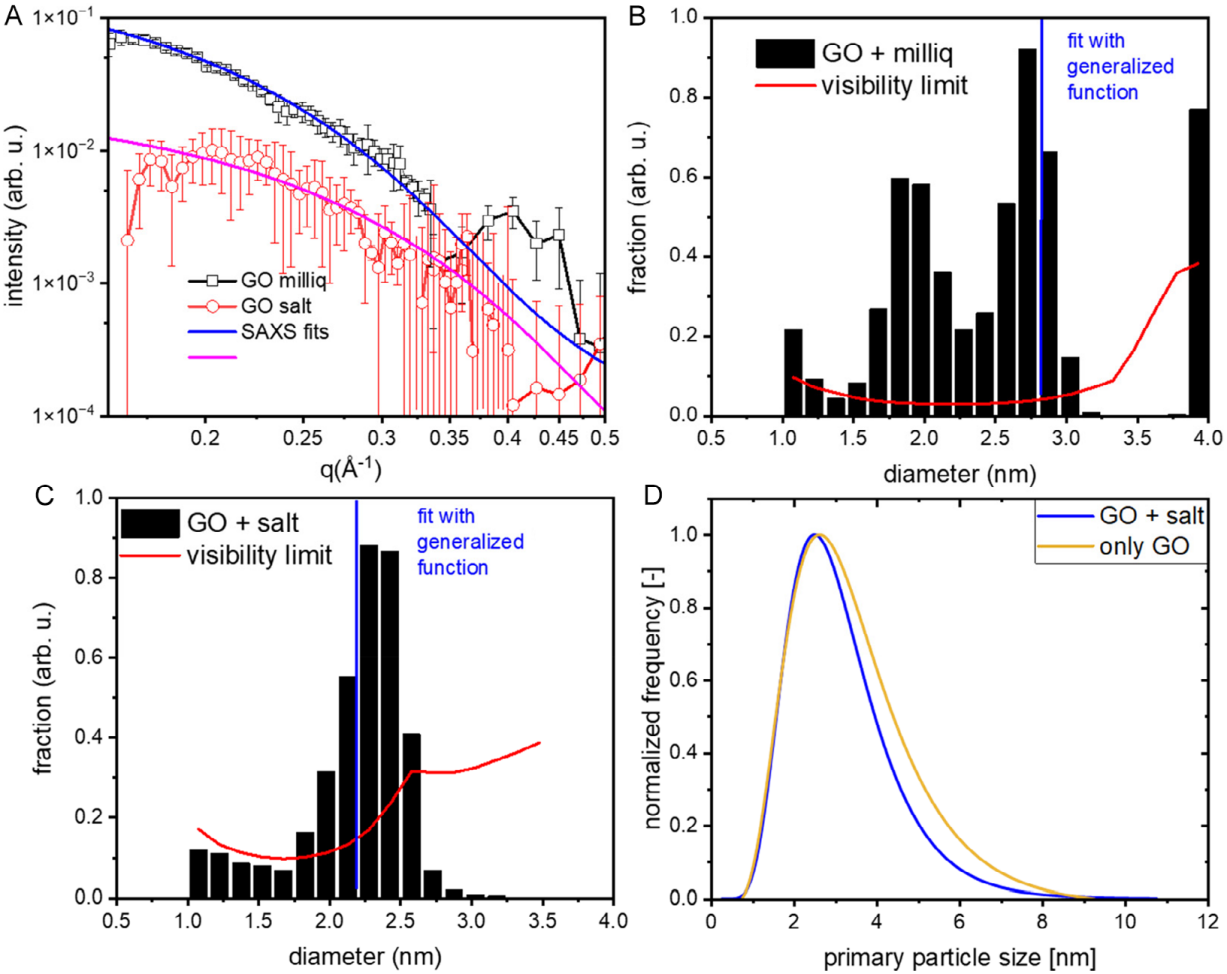
(A) SAXS data on the formed fragments after irradiation with 400 nm, 1ps laser pulses, averaging over delays from 3 to 20 µs. The lines are fitted with a generalized SAXS function resulting in particles with mean sizes of 2.8 +/− 0.1 nm and 2.2 +/− 0.3 nm for Au/GO composite particles without salt (MilliQ water) and Au/GO composite particles with 200 mM NaCl, respectively. (B) and (C) represents histograms of the particle size distribution from fitting the data in (A) by a rMC approach of freely varied fraction of spherical particles in the interval between 1 and 4 nm. The red line shows the visibility limit below which the derivation of fractions is not significant. (D) represents the particle size distribution (lognormal Fits) of the same samples stemming from post‐mortem TEM analysis (see Figure S11 for Histograms).

As a result, although the peak maximum shows only a slightly lower mean particle size for the particles fragmented in MilliQ water, the sample fragmented in MilliQ water also results in a bimodal size distribution with a significant number of particles bigger than 4 nm, whereas the fragmentation made in 200 mM NaCl solution barely shows any particles bigger than 4 nm. Additionally, we performed time‐resolved SAXS measurements where we compared the fragmentation of the same adsorbed AuNP on GO (25 wt% mass coverage, but with 0.3 g/l GO) in either the NaCl solution or in pure water. The SAXS data (Figure 4A) at a delay of 3–20 µs after fragmentation with a 1 ps laser pulse at 400 nm leads to average fragment sizes of 2.2 +/− 0.3 nm in salt, while the same fragmentation leads to 2.8 +/− 0.1 nm fragments in neat water. Additionally, Figure 4B,C display the result from an analysis of the SAXS measurement with a rMC approach, showing a peaked size distribution around 2 nm in salt, which broadens without salt and shifts to larger values. Caution should be given to the rMC results, as they only represent a possible realization of size distributions. At the same time, we have reported earlier that during fragmentation of AuNPs in the presence of GO without the preadsorption on GO and without salt addition, the fragment size reaches about 2.7 +/− 0.16 nm, a similar value as here in neat water [26]. To compare those in‐situ SAXS results with the TEM results in this paper, we also conducted post‐mortem TEM measurements using the SAXS samples. As a result, the distance in particle size is still measurable with the 200 mM NaCl sample yielding a particle size of 3.1 +/− 1.3 nm and the neat water sample yielding a particle size of 3.5 +/− 1.6 nm (see Figure 4D).

As a result, those results show that (a) fragment size is sensitive to the salt addition, which reveals a competition of agglomeration and re‐adsorption, and (b) at this high salt concentration, the re‐adsorption is triggered in the same way as during the adsorption of the initial particles already within microseconds. Therefore, the addition of 200 mM NaCl seems to be a crucial step to ensure rapid adsorption of the Au clusters onto the GO sheets after fragmentation, but can also lead to increased agglomeration/aggregation of the clusters. To safely avoid such effects, the GO sheet distance should be kept minimal (~15 µm).

We also acknowledge that the model of GO being present in suspension as a flat sheet may be too trivial. To the best of our knowledge, the geometry and general condition of GO sheets in water are barely investigated. Our results could point toward a more complex scenario than simply assuming a sheet‐like geometry in water. First, as the GO sheets can float freely, the calculated mean distance of the sheets can be significantly smaller for some GO sheets or similarly in a local wrinkled geometry. Additionally, in an earlier study, we already described that Au/GO composites fabricated via the same method flocculate and sediment over the course of a few hours [27]. Therefore, it is possible that a small degree of agglomeration of the pre‐loaded sheets was also present during the laser fragmentation. But in general, our results clearly show that GO can exhibit an important stabilization effect on gold fragments that form during LFL, when performed under conditions of high salinity and with initial AuNPs being preabsorbed. While the microsecond‐resolved SAXS results reveal that fragment stabilization by GO adsorption does involve a fast component, which quenches the sizes on the microsecond scale, the variation of GO sheet distance shows that interaction across distant sheets by desorption and re‐adsorption processes and/or GO sheet interaction happens on a millisecond time scale and beyond.

## 4 Conclusion

In this publication, we investigated the possibility to achieve size quenching during laser fragmentation in liquid by the addition of support material during the laser process. This method has already been described in the literature for laser ablation. For laser fragmentation, a previous study of ours showed that a simple mixing of initial ~60 nm AuNPs and GO sheets as support material before laser fragmentation did not lead to a size quenching [26]. In fact, the resulting particles were larger than the ones where no GO was added. Therefore, we first adsorbed ~60 nm AuNPs onto the thin GO sheets prior to laser fragmentation and then fragmented the composite with single laser pulses. Because of the close spatial proximity between GO sheets and the initial Au particles and the addition of 200 mM NaCl, the created Au clusters are forced to adsorb onto the GO sheets directly after the laser fragmentation, which stops the coalescence of the particles, leading to a reduced particle size. To study the influences that lead to a reduced particle size, we conducted two sets of experiments. First, the variation of the initial AuNP mass loading on the GO sheets allowed us to investigate the effects of Au cluster density generated during the laser irradiation (dependent on AuNP concentration and their spatial closeness) on the coalescence of the clusters. Second, the alteration of the GO sheet distance (dependent on GO concentration) allowed us to investigate a spatial distance that the generated clusters need to overcome before adsorption, and what effect the distance has on the coalescence of the clusters. This second experiment is especially important as the salt‐induced reabsorption is a barrierless and instantaneous process, which is expected to be the same in every sample, but the distance that the clusters need to overcome to reach the next GO sheet is crucial to impede agglomeration/aggregation effects accelerated by the high ionic strength.

By combining the results of both sets of experiments, we were able to identify experimental parameters, which lead to a minimal particle size: First, the overall concentration of the initial AuNPs has to be quite low (below ~3 mg/L) with a small amount of agglomerated initial particles on the GO sheets (mass loading ideally smaller than 10 wt%) to keep the cluster density during the fragmentation low. Second, the GO concentration should be chosen in a way that the sheet–sheet distance fits to the diffusion speed of the clusters to ensure spatial closeness to the generated Au clusters and a fast immobilization of Au clusters. As the Au clusters diffuse with a diffusion speed of ~15 µm/s, a sheet distance around or even below 15 µm would be optimal. From our current results, using a GO concentration much below this value does not seem favorable, as the increased optical density can lead to significant laser light attenuation. A minimal particle size is only achieved if both conditions are fulfilled.

Please note that in addition to the mass transport via diffusion processes, at the current stage, the role of any translational movement of the clusters that are ejected during the laser‐induced phase explosion [8, 9, 31], (of the fragmenting gold particles) remains elusive and cannot yet be accounted for. Further joint experimental (ex and in situ) and theoretical (e.g. via atomistic MD simulation) [31] studies are needed to unequivocally answer this question.

In summary, to minimize the coalescence of Au clusters after laser fragmentation, both the concentration and dispersion of the initial AuNPs and the concentration of the GO sheets must be coordinated with each other. As an outlook, this new approach may be used for mechanistical investigations of the post‐synthesis growth processes during surfactant‐free laser‐based synthesis of nanoparticles. Especially by extending the sample matrix during the in‐situ SAXS experiments (different mass loadings and GO sheet distances), diffusion times of the Au clusters could be analyzed in detail. Although this study represents a proof‐of‐concept rather than a process and material optimization, we can already provide a first brief outlook concerning the applicatory implications of this study. In heterogeneous catalysis, the desired mass loadings (of nanoparticles on support) do significantly vary from case to case. As a rule of thumb, only a few or even less wt% are often typical in applications related to thermal catalysis [34], while some tens of wt%, e.g. are common in electrocatalysis with Pt/C [35]. In this study, we have observed a better stabilization effect of the support when the mass loading was considerably low, hence favoring applications in thermal catalysis. So far, we have not shown transferability to other supports common to thermal catalysis, e.g. TiO_2_, Al_2_O_3_, SiO_2_, but transferability is likely given that diffusion‐control is an important factor during the size quenching mechanism.

## Supporting Information

Additional supporting information can be found online in the Supporting Information section. **Supporting Fig. S1**: Comparison of the measured UV–vis spectra of the Au/GO samples with different mass loading compared to the simulated ones determined from fitting an additive linear combination of the initial colloidal AuNPs and GO (used for the preparation of Au/GO) to the measured data while varying the relative concentrations of the AuNP and GO. The fitting procedure has been done via minimization of the squared sum error within the spectral range of 200 nm to 600 nm using the SOLVER tool in Microsoft Excel. The fitted results represent a (mostly) agglomerate free state of the AuNP that is comparable to the initial AuNP colloid used during the barrierless self‐assembly of AuNPs onto GO in line with a previous study [1]. **Supporting Fig. S2**: OCT measurement setup for determination of the water layer thickness in the Flat Jet. The measuring position is the same as the position used for laser beam entrance during the fragmentation experiments. (A) Schematic and (B) Photographed setup and (C) exemplary picture of a scan. **Supporting Fig. S3**: Exemplary TEM pictures of ~60 nm Au/GO with (A) 5 wt%, (B) 10 wt% and (C) 25 wt%. **Supporting Fig. S4**: (A) Distribution of the relative number of agglomerates in dependance of their agglomerate size for the different gold mass loadings on the GO sheets and (B) same distribution but for the number of particles within the agglomerates for the different agglomerate sizes. For the measurement, several hundred adsorbed AuNPs were classified on whether they were adsorbed as a single particle or as an agglomerate. **Supporting Fig. S5**: TEM pictures after LFL experiments of the Au/GO samples with different mass loadings and the LFL of AuNPs in pure MilliQ water. **Supporting Fig. S6**: Histograms (brown bars) and cumulative counts (black lines) extracted from the TEM images to create the lognormal Fits in figure 2B. **Supporting Fig. S7**: TEM pictures after LFL experiments of the Au/GO samples with 25 wt% and different Au/GO concentrations. **Supporting Fig. S8**: Histograms (brown bars) and cumulative counts (black lines) extracted from the TEM images to create the lognormal Fits in figure 3A. **Supporting Fig. S9**: Calculated mean distance of GO sheets depending on the GO concentration. **Supporting Fig. S10**: A and B show histograms (brown bars) and cumulative counts (black lines) extracted from TEM images to create the lognormal Fits in C. Sample: Au/GO at 25 wt% (10.7 mg/L Au/GO) suspended in 200 mM NaCl or MilliQ water. **Supporting Fig. S11**: A and B show histograms (brown bars) and cumulative counts (black lines) extracted from the TEM images to create the lognormal Fits for Figure 4D. The samples were produced during the in situ SAXS experiments. **Supporting Fig. S12**: Raman measurements of GO, before and after single‐pulse LFL, showing no significant change in the spectra and therefore no significant change of the GO structure from laser irradiation can be observed. **Supporting Table S1**: Used volumes and mass concentration of the different components during the Au‐on‐GO supporting process. In all cases, an inclined blade stirrer with a rotation speed of 700 rpm was used. For the supporting process, the listed amounts of NaCl and GO were dispersed in MilliQ water and stirred at 700 rpm before the Au colloid was slowly dropped in using a syringe pump yielding the resulting total volume of 400 or 150 mL Au/GO composite, respectively. **Supporting Table S2**: Optically‐determined concentrations of AuNPs and GO from linear fitting of the UV–vis spectrum (200 –600 nm) in Figure S1 with the UV–vis spectrum (and known concentrations) of the initial colloidal AuNPs and GO used for the preparation of Au/GO. The optically‐determined gold mass loading was calculated from the respective concentrations. All optically‐determined values mainly trace the amount of primary AuNPs within the analyzed, respective Au/GO sample. **Supporting Table S3**: Technical details of the OCT measurement system. **Supporting Table S4**: Calculation of the maximal number of 2 nm clusters which can be generated from the initial AuNPs adsorbed on 4 different 2 × 2 µm^2^ GO sheet areas. **Supporting Table S5**: Calculation of the GO sheet distance. Fixed values. **Supporting Table S6**: Calculation of the GO sheet distance. Sample values.

## Funding

This study was supported by Deutsche Forschungsgemeinschaft (Grants 491072288, 491072288).

## Conflicts of Interest

The authors declare no conflicts of interest.

## Supporting information

Supplementary Material

## Data Availability

The data that support the findings of this study are available from the corresponding author upon reasonable request.
