# Supplementary material for: Inhibition of Particle Growth During Single‐Pulse Laser Fragmentation by Barrierless Adsorption of the Just‐Formed Gold Nanoparticles on Graphene Oxide
Source: Chemphyschem. 2026 Mar 3;27(5):e202500671. doi: 10.1002/cphc.202500671 (PMC12956156; doi:10.1002/cphc.202500671)
Supplement: Supplementary file 1 — Supplementary Material [file CPHC-27-e202500671-s001.pdf]

Supporting Information

Inhibition of particle growth during single-pulse laser fragmentation by barrierless adsorption of the just-formed gold nanoparticles on graphene oxide

Meike Tack[a], Anton Plech[b], Yogesh Pokhrel[b], Maron Dolling[c,d], Martin Ahrens[c,e], Gereon Hüttmann[c,d,e], Sven Reichenberger*[a]

[a] M. Tack, Dr. habil. S. Reichenberger
Technical Chemistry 1
University Duisburg-Essen
Universitätsstraße 7
E-mail: sven.reichenberger@uni-due.de

[b] PD Dr. A. Plech, Y. Pokhrel
Institute for Photon Science and Synchrotron Radiation
Karlsruhe Institute of Technology
Hermann-von-Helmholtz Platz 1
76344 Eggenstein-Leopoldshafen, Germany

[c] Maron Dolling, Martin Ahrens, Prof. Gereon Hüttmann
Institute for Biomedical Optics
University of Lübeck
Peter-Monnik-Weg 4
23562 Lübeck, Germany

[d] Maron Dolling, Prof. Gereon Hüttmann
Medical Laser Center Lübeck GmbH
Peter-Monnik-Weg 4
23562 Lübeck, Germany

[e] Martin Ahrens, Prof. Gereon Hüttmann
German Center for Lung Research (DZL)

S1: Determination of the gold concentration in Au/GO particle dispersions used for LFL

The ~60 nm gold nanoparticles (AuNPs) (that were obtained from laser-generated AuNP colloids via step-wise centrifugation) were adsorbed onto the graphene oxide (GO) sheets via barrierless self-assembly method, in line with our previous study [1]. Note that the colloidal gold nanoparticles used in our study are solely electrostatically stabilized by their zeta potential < -30 mV without using any additional steric ligands or organic additives (like citrate). During the adsorption method, the colloidal AuNPs were slowly added into a stirred volume of colloidal GO which contained 200 mM NaCl to screen the electrostatically stabilizing charges on the AuNPs' surface and induce the barrierless self-assembly (adsorption of AuNPs on GO). In the present study, higher mass loadings of AuNPs on GO were

achieved by adding the increasing volumes of AuNP colloid to a decreasing volume of the colloidal GO dispersion yielding 400 and 150 mL total volume, respectively (see table S1). The obtained Au/GO dispersions were characterized via UV-VIS spectroscopy to determine the concentrations of gold and GO in the sample. As a reference, we used the UV-VIS spectra and concentrations of the initial ~60 nm AuNPs (31.4 mg/L) and the gold-free (initial) GO dispersion (20.0 mg/L). The concentration of the initial gold colloid was determined from a calibration curve from literature (UV-Vis spectra from differently concentrated, laser-generated colloidal AuNPs [2] while the concentration of the GO dispersion was known from the mass of dry GO and added volume of water that were used during the preparation of the colloidal dispersion. To determine the concentrations of adsorbed AuNP and GO in each Au/GO dispersion we used the linear combination $A_{GO+Au}(\lambda) = x_{Au} \cdot A_{Au}(\lambda) + x_{GO} \cdot A_{GO}(\lambda)$ where $A_{Au}(\lambda)$ and $A_{GO}(\lambda)$ are the UV-VIS spectra of the initial gold and GO colloids (see Fig. S1) while x_{Au} and x_{GO} represent relative concentrations of Au and GO with respect to the initial concentrations of AuNPs ($c_{Au,0}$ =31.4 mg/L) and GO ($c_{GO,0}$=20.0 mg/L). By minimizing the mean square error between experimental UV-VIS data in Fig. S1 and the linear combination under variation of x_{Au} and x_{GO} the linear combination was fitted to the experimental data within the spectral region between 200 nm and 600 nm. With the determined relative concentrations of Au (x_{Au}) and GO (x_{GO}) and the known initial concentrations $c_{Au,0}$ and $c_{GO,0}$ the absolute concentrations of the adsorbed AuNPs ($c_{Au} = c_{Au,0} \cdot x_{Au}$) and GO ($c_{GO} = c_{GO,0} \cdot x_{GO}$) in the dispersed Au/GO samples has been obtained and summarized in Table S1. Additionally, the mass loading of AuNPs on GO was determined via $wt_{Au} = \frac{c_{Au}}{c_{Au}+c_{GO}} \cdot 100\%$ and tabulated in Table S2. In the first part of the paper (Fig 1), we also included results from an Au/GO composite with 2 wt%. But because of the small amount of AuNPs in this sample, we could not verify the Au and GO concentrations in the sample with the presented method, so we excluded this sample from the LFL experiments.

Please note that we specifically limited this analysis procedure to the spectral range below 600 nm in order to only characterize the concentration of AuNPs and GO within the Au/GO sample that obtain a low degree of agglomeration. Consequently, this analysis specifically targets the Au/GO sheets that contain well-dispersed AuNPs. These are specifically relevant for the LFL experiment which compares to the established case (in literature) of an LFL of colloidal AuNPs (without GO support)[3, 4]. From Table S2, the nominal mass load (set by the used volumes of gold and GO colloid) and the mass load from this UV-VIS analysis are in very good agreement for the Au/GO samples indicating that the AuNP are

well dispersed on the GO sheets. In turn, the optical readout of the 35 wt% sample only included 70% (24.7 wt% instead of 35 wt%) of the nominal mass load indicating that this sample contained about 30% of agglomerated AuNPs.

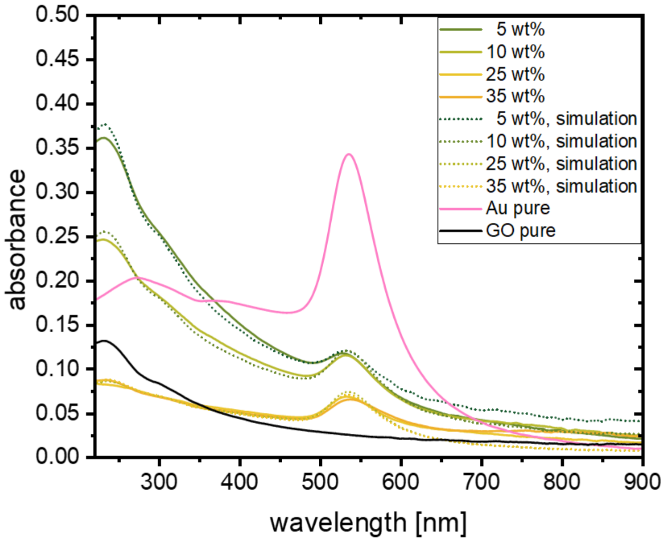

Figure S1: Comparison of the measured UV-Vis spectra of the Au/GO samples with different mass loading compared to the simulated ones determined from fitting an additive linear combination of the initial colloidal AuNPs and GO (used for the preparation of Au/GO) to the measured data while varying the relative concentrations of the AuNP and GO. The fitting procedure has been done via minimization of the squared sum error within the spectral range of 200 nm to 600 nm using the SOLVER tool in Microsoft Excel. The fitted results represent a (mostly) agglomerate free state of the AuNP that is comparable to the initial AuNP colloid used during the barrierless self-assembly of AuNPs onto GO in line with a previous study[1].

Table S1: Used volumes and mass concentration of the different components during the Au-on-GO supporting process. In all cases, an inclined blade stirrer with a rotation speed of 700 rpm was used. For the supporting process, the listed amounts of NaCl and GO were dispersed in MilliQ water and stirred at 700 rpm before the Au colloid was slowly dropped in using a syringe pump yielding the resulting total volume of 400 or 150 mL Au/GO composite, respectively.

Nominal mass loading [wt%]	Total volume of final composite [mL]	Added NaCl volume (4 M) [ml]	Added GO volume (1 g/L) [mL]	Added Au colloid volume [mL]	Dripping speed of the Au colloid [mL/min]
5	400	20	13	11 (60 mg/L)	1.0
10	400	20	13	29 (70 mg/L)	1.3
25	400	20	13	73 (60 mg/L)	1.2
35	150	7.5	5	43 (60 mg/L)	1.1

Table S2: Optically-determined concentrations of AuNPs and GO from linear fitting of the UV-VIS spectrum (200 nm – 600 nm) in Fig. S1 with the UV-VIS spectrum (and known concentrations) of the initial colloidal AuNPs and GO used for the preparation of Au/GO. The optically-determined gold mass loading was calculated from the respective concentrations. All optically-determined values mainly trace the amount of primary AuNPs within the analyzed, respective Au/GO sample.

Nominal mass loading [wt%]	Optically-determined Au-concentration [mg/L]	Optically-determined GO-concentration [mg/L]	Optically-determined gold mass loading [wt%]
5	2.4	52.8	4.3
10	3.6	32.3	10.1
25	2.9	7.8	27.3
35	2.8	8.4	24.7

S2: Determination of the water layer thickness of the Flat Jet

To ensure single-pulse conditions during the fragmentation experiments, several experimental parameters had to be matched, such as the flow rate of the colloidal solution, the height of the laser beam and the repetition rate of the laser [5]. To convert the flow rate of the colloidal solution from volume per second to length per second, the thickness of the Flat Jet layer at the point of laser beam entrance must be determined. Optical coherence tomography (OCT) was used for this determination. This non-invasive imaging technique is normally used in the medical field, especially for eye exams, and creates 3D images of tissue with high resolution [6]. Using a specially designed probe with microscopic resolution normally used for clinical studies, we were able to measure the thickness of the flat jet at the same position as the laser beam entrance during the fragmentation experiment without touching, and therefore disturbing, the water layer (see Figure S2). For more detailed information regarding OCT measurements, please see this literature[7] for general information and this literature [8] for detailed information about the OCT setup which was used in the presented experiment.

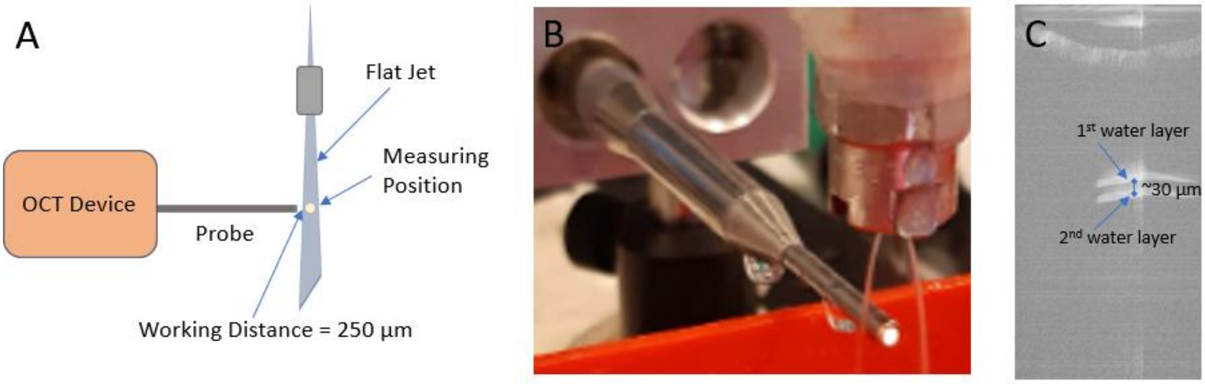

Figure S2: OCT measurement setup for determination of the water layer thickness in the Flat Jet. The measuring position is the same as the position used for laser beam entrance during the fragmentation experiments. (A) Schematic and (B) Photographed setup and (C) exemplary picture of a scan.

Table S3: Technical details of the OCT measurement system.

Wavelength	λ = 750 ± 200 nm
Working Distance / water	f = 250 µm
Rayleigh-length	z_r = 290 µm
Numerical Aperture	NA = 0.17
Axial Resolution	d_z = 1.16 µm
Axial scanning area / water	FOV ~ 540 µm
Lateral Resolution	d_x = 2.15 µm
Lateral scanning area / water	FOV ~ 600 µm
Endoscope	Custom made GRIN lens system: Focus lens: 49-305, Edmund Optics, U.S.), relay lens: LFRL-180-025-20, length 26.556 mm, focus lens: LFRL-180-025-50, length 5.252 mm (Grintech GmbH, Jena, Germany)
Beamsplitter	TW630R5A2 (Thorlabs GmbH, Bergkirchen, Germany)
Laser	SuperK Extreme EXW-4 OCT (NKT PHOTONICS A/S, Birkerød, Denmark)
Spectrometer	custom-built high-speed spectrometer (Thorlabs GmbH, Bergkirchen, Germany)
Camera	Piranha 4, Teledyne DALSA Inc., Canada, 100 kHz A-scan rate
Probe	Thorlabs OCT-H 900

S3: Agglomeration of AuNPs on GO support before LFL

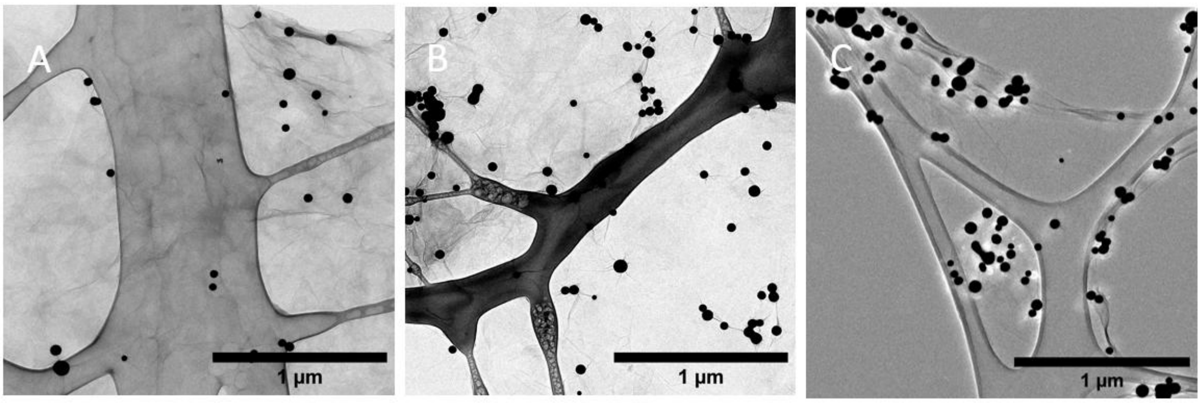

Figure S3: Exemplary TEM pictures of ~60 nm Au/GO with (A) 5 wt%, (B) 10 wt% and (C) 25 wt%.

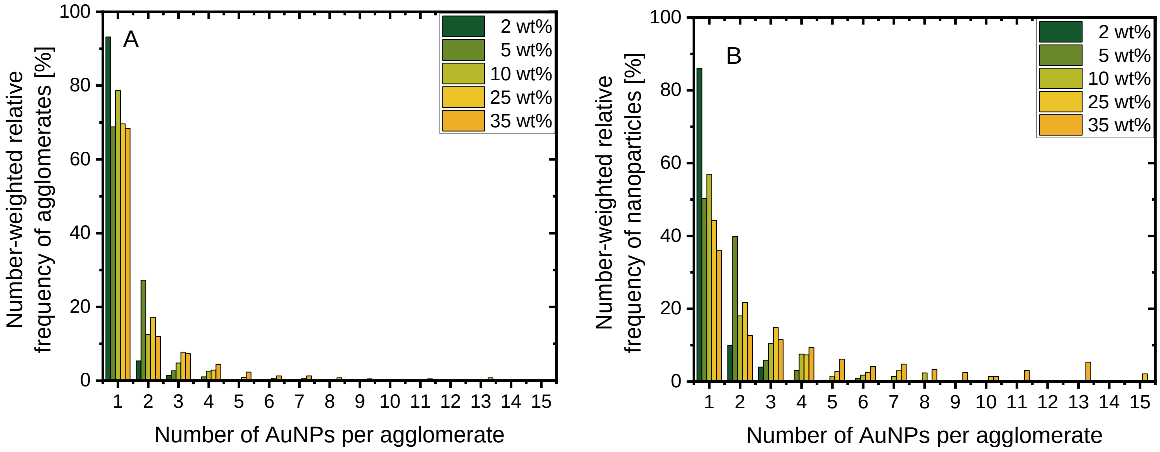

Figure S4: (A) Distribution of the relative number of agglomerates in dependance of their agglomerate size for the different gold mass loadings on the GO sheets and (B) same distribution but for the number of particles within the agglomerates for the different agglomerate sizes. For the measurement, several hundred adsorbed AuNPs were classified on whether they were adsorbed as a single particle or as an agglomerate.

S4: Calculation of cluster quantity

Calculation of cluster quantity:

Figure 1 shows that the overall number of initial AuNPs and their degree of agglomeration on the 4 exemplary examined 2 x 2 µm^2 areas within the shown the GO sheets depends on the gold mass loading. This means that during the laser irradiation of the same GO sheet area a different volume of initial AuNPs is fragmented which results in a different number of generated clusters. To calculate this maximum number of generated clusters we proceeded the following way. First, we measured the footprint of the initial ~60 nm AuNPs adsorbed on those 4 exemplary examined 2 x 2 µm^2 GO sheet areas and calculated the total surface of those initial AuNPs. Then we used these results to calculate the total volume of all adsorbed initial particles. By assuming that all of the initial particles are being transformed into clusters and by assuming a cluster size of 2 nm, the maximum number of generated clusters can be calculated for both mass loadings (10 and 35 wt%) (see Table S4).

Table S4: Calculation of the maximal number of 2 nm clusters which can be generated from the initial AuNPs adsorbed on 4 different 2x2 µm^2 GO sheet areas.

Mass loading [wt%]	Measured footprint of initial AuNPs [µm^2]	Number of initial AuNPs	Calculated total volume of initial AuNPs [µm^3]	Max. number of generated 2 nm clusters
5	0.358	141	0.014	3.4E+6
10	0.728	303	0.027	6.4E+6
25	0.748	254	0.031	7.3E+6
35	0.945	254	0.069	1.04E+7

S5 TEM results of Au/GO composites and the MilliQ sample after LFL

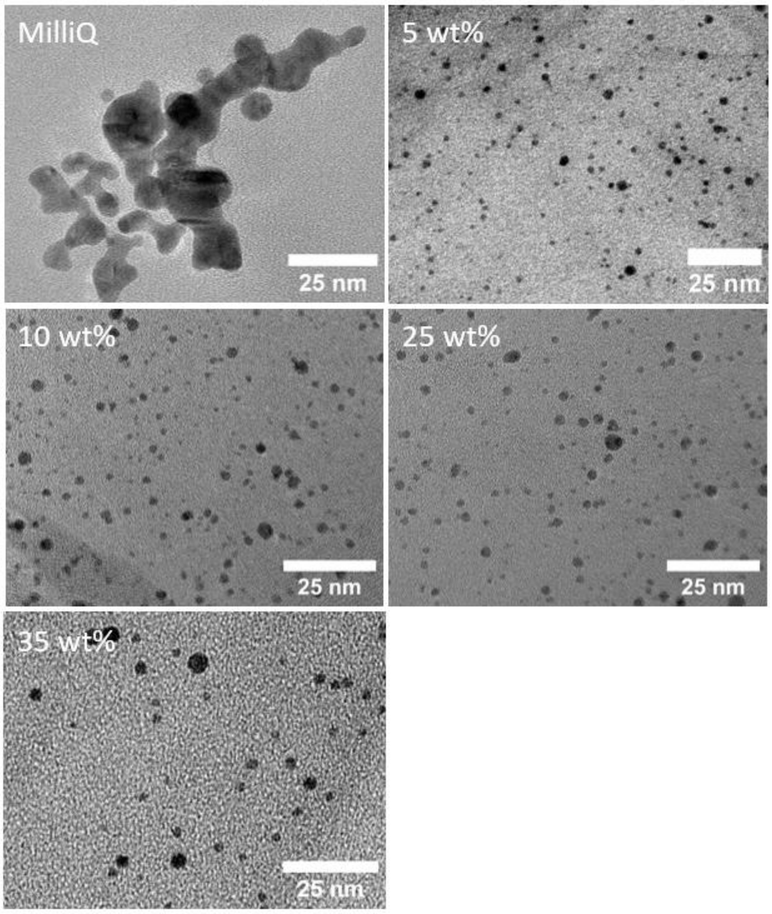

Figure S5: TEM pictures after LFL experiments of the Au/GO samples with different mass loadings and the LFL of AuNPs in pure MilliQ water.

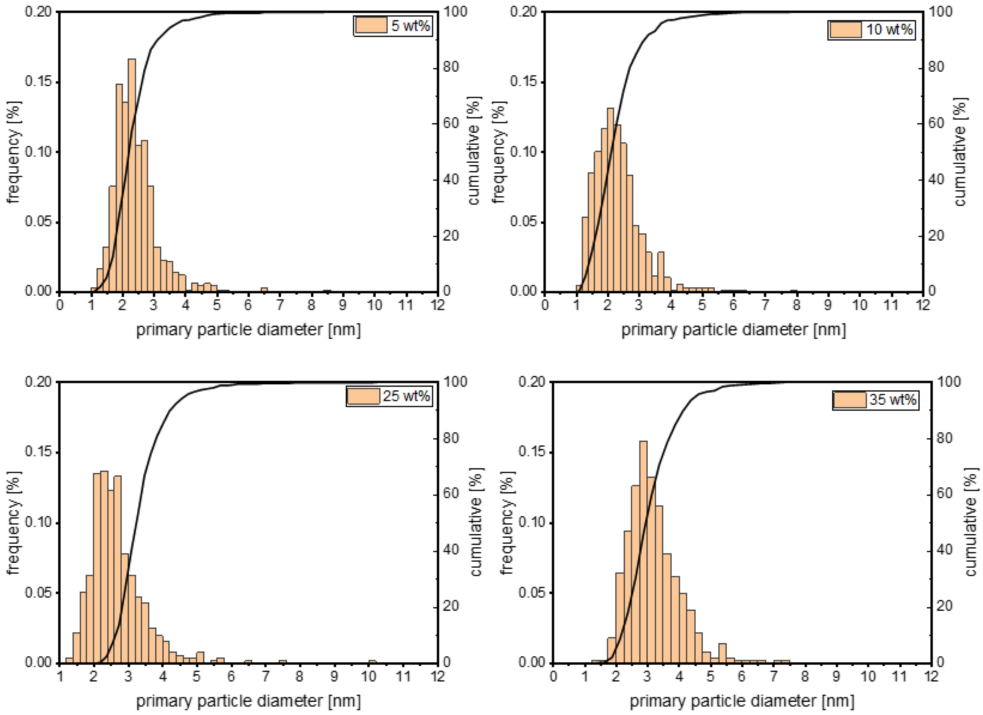

Figure S6: Histograms (brown bars) and cumulative counts (black lines) extracted from the TEM images to create the lognormal Fits in figure 2B.

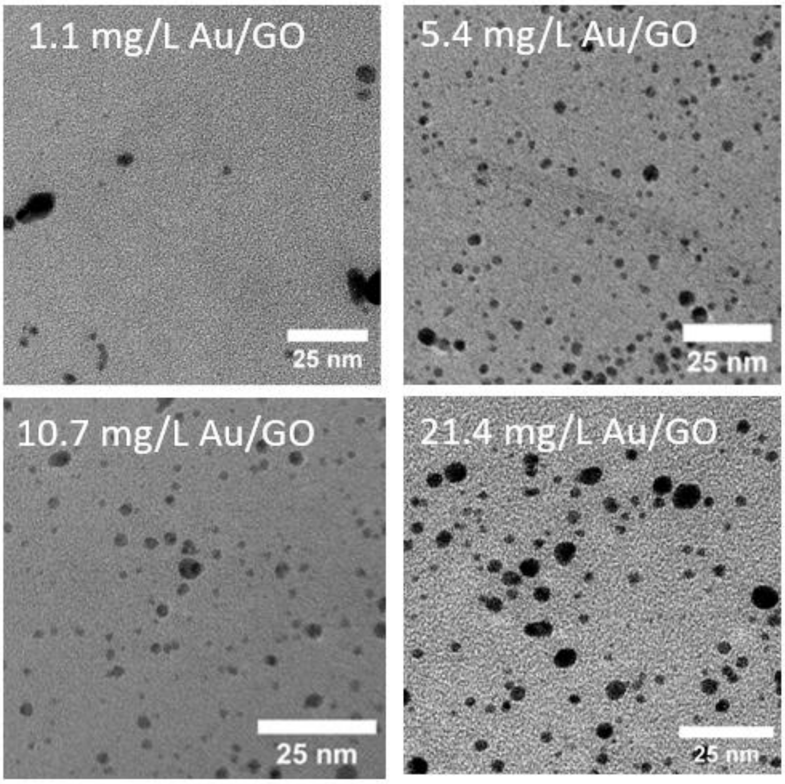

Figure S7: TEM pictures after LFL experiments of the Au/GO samples with 25 wt% and different Au/GO concentrations.

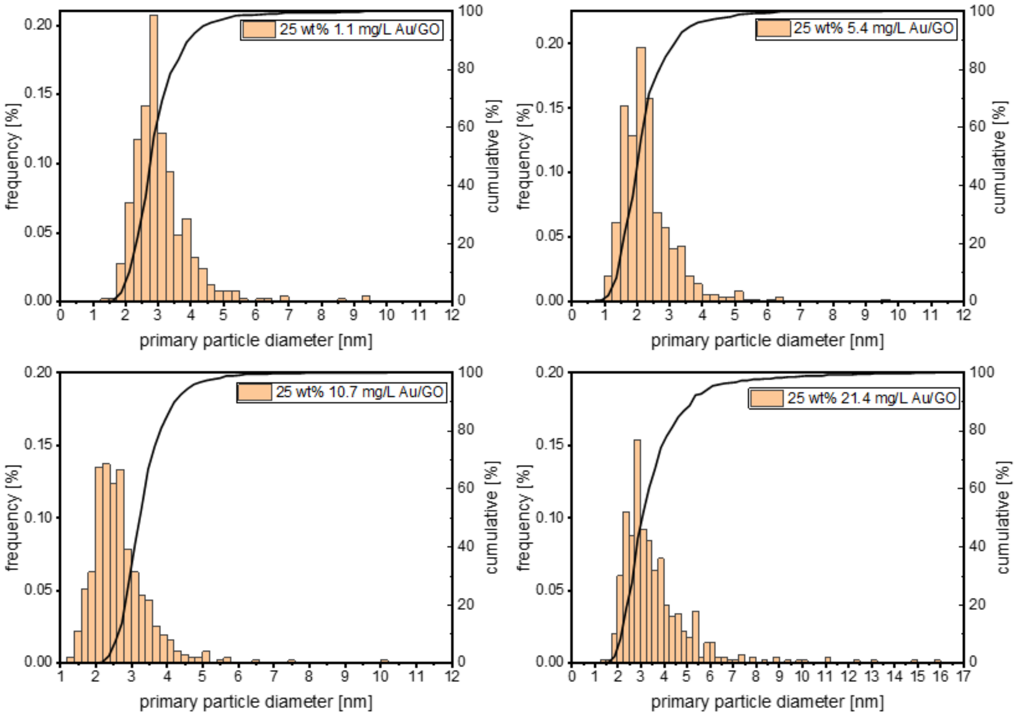

Figure S8: Histograms (brown bars) and cumulative counts (black lines) extracted from the TEM images to create the lognormal Fits in figure 3A.

S6 Calculation of GO sheet distance:

To calculate the distance of the GO sheets in our samples, we assume that the liquid sample is in a simulated cuboid and the GO sheets are stacked onto each other with equal distances. We define that the floor area of the cuboid is 2 x 2 μm^2 with a volume of 1 ml. From this, we can calculate the height of the cuboid. By assuming that every GO sheet covers the floor area and with the materials density (which we measured ourselves by using a pycnometer) and assuming perfect exfoliation of the material down to monolayers with 1.1 nm thickness, we can calculate the mass per GO sheet. Now we can determine the number of GO sheets in the simulated cuboid by multiplying the GO concentration present in the sample with the volume of the simulated cuboid (which gives us the absolute mass of GO) divided by the mass of a single GO sheet. In the last step, we take the height of the cuboid and divide it through the calculated number of GO sheets to receive a mean distance of the GO sheets, assuming that they are stacked with equal distances.

Table S5: Calculation of the GO sheet distance. Fixed values.

Floor area [μm^2]	Cuboid volume [mL]	Height of cuboid [cm]	GO conc. [mg/L]	# of sheets per layer	Thickness of monolayer GO [nm]	Density of GO sheets [g/mL]	Mass per GO sheet [g]
4	1	2.5E7	52.8	1	1.1 [9]	1.14	5E-15

Table S6: Calculation of the GO sheet distance. Sample values.

Mass loading [wt%]	Gold conc. [mg/L]	GO conc. [mg/L]	# of stacked GO sheets	Distance of GO sheets [µm]
5	2.4	52.8	1.1E10	23.4
10	3.6	32.2	6.4E9	39.0
25	2.9	7.8	1.6E9	160.8
35	2.8	8.4	1.7E9	149.3
25	0.3	0.8	1.6E8	1607.7
25	1.5	3.9	7.8E8	321.5
25	5.8	15.6	3.1E9	80.4

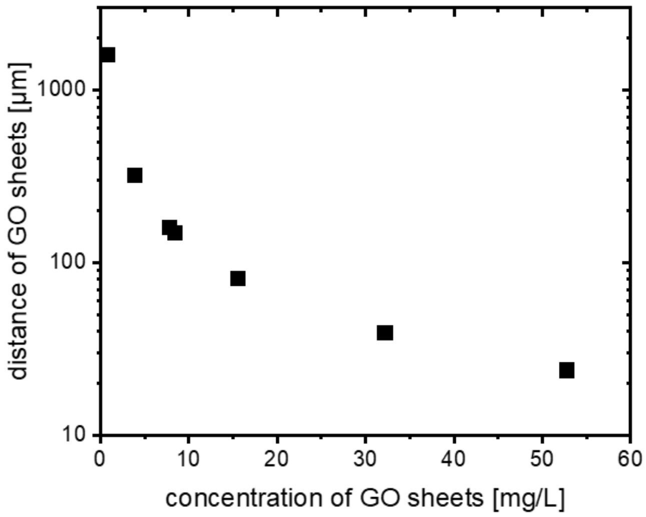

Figure S9: Calculated mean distance of GO sheets depending on the GO concentration.

S7 Comparison of fragmentation in MilliQ water or 200 mM NaCl solution

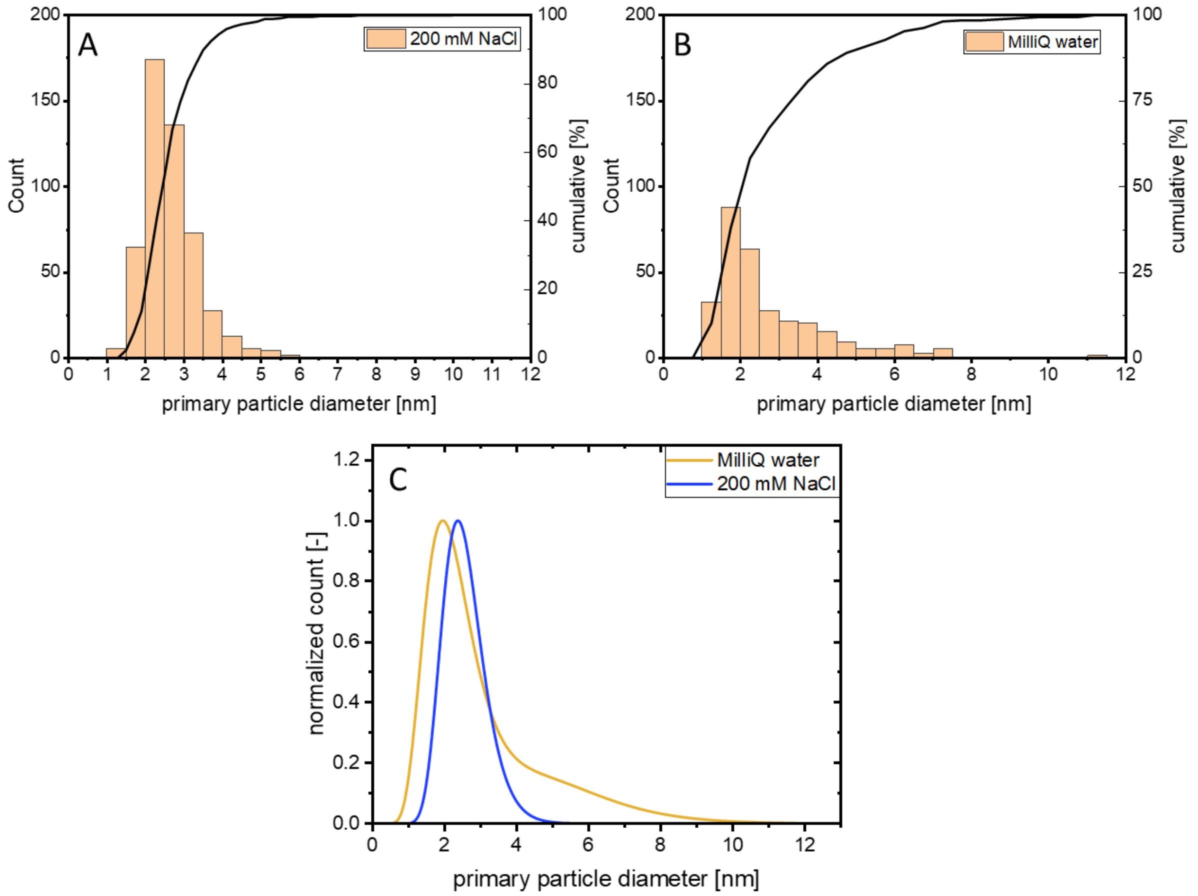

Figure S10: A and B show histograms (brown bars) and cumulative counts (black lines) extracted from TEM images to create the lognormal Fits in C. Sample: Au/GO at 25 wt% (10.7 mg/L Au/GO) suspended in 200 mM NaCl or MilliQ water.

S8 Post mortem TEM analysis of SAXS samples

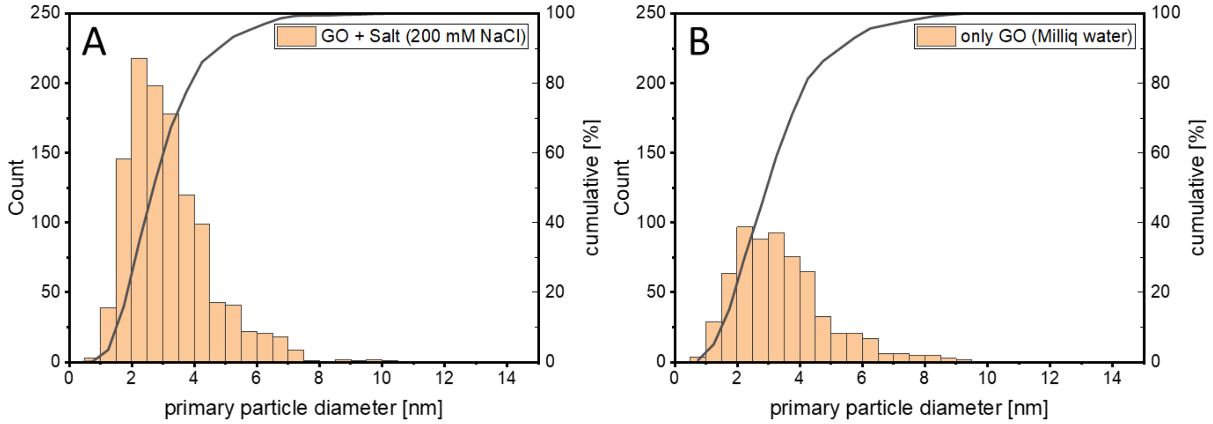

Figure S11: A and B show histograms (brown bars) and cumulative counts (black lines) extracted from the TEM images to create the lognormal Fits for Figure 4D. The samples were produced during the in-situ SAXS experiments.

S9 Raman Characterization of the GO sheets

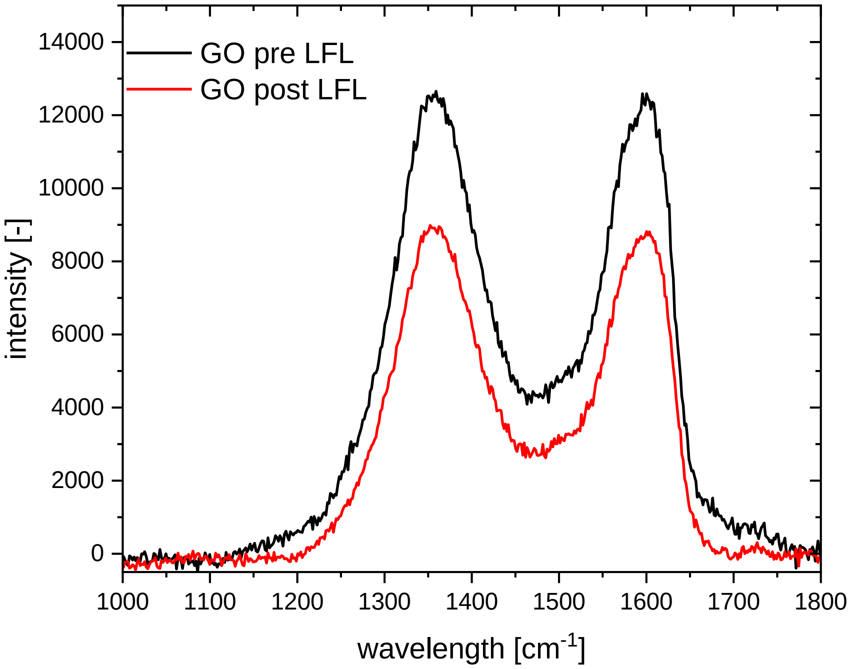

Figure S12: Raman measurements of GO, before and after single-pulse LFL, showing no significant change in the spectra and therefore no significant change of the GO structure from laser irradiation can be observed.

The authors have cited additional references within the Supporting Information.

References

[1] M. Tack; A. Plech; S. Reichenberger. Adsorption of Colloidal Nanoparticles to Supports: Employing Electrostatic Charge Screening and Barrierless Self-Assembly as Nanointegration Strategy. *Chemphyschem* **2024**, e202400121.

[2] L. Gamrad. Rationelles Design und biomedizinische Anwendung von multifunktionalen Goldnanopartikel-Biokonjugaten. Dissertation; Universitätsbibliothek Duisburg-Essen, Duisburg, Essen, 2017.

[3] A. R. Ziefuß; S. Barcikowski; C. Rehbock. Synergism between Specific Halide Anions and pH Effects during Nanosecond Laser Fragmentation of Ligand-Free Gold Nanoparticles. *Langmuir* **2019,** *35* (20), 6630–6639.

[4] A. R. Ziefuss; S. Reich; S. Reichenberger; M. Levantino; A. Plech. In situ structural kinetics of picosecond laser-induced heating and fragmentation of colloidal gold spheres. *Phys. Chem. Chem. Phys.* **2020,** *22*, 4993.

[5] A. R. Ziefuß; S. Reichenberger; C. Rehbock; I. Chakraborty; M. Gharib; W. J. Parak; S. Barcikowski. Laser Fragmentation of Colloidal Gold Nanoparticles with High-Intensity Nanosecond Pulses is Driven by a Single-Step Fragmentation Mechanism with a Defined Educt Particle-Size Threshold. *J. Phys. Chem. C* **2018,** *122* (38), 22125–22136.

[6] S. Aumann; S. Donner; J. Fischer; F. Müller. *High Resolution Imaging in Microscopy and Ophthalmology: New Frontiers in Biomedical Optics*. *Optical Coherence Tomography (OCT): Principle and Technical Realization*: Cham (CH), 2019.

[7] W. Drexler; J. G. Fujimoto. *Optical Coherence Tomography;* Springer International Publishing: Cham, 2015.

[8] H. Schulz-Hildebrandt; M. Pieper; C. Stehmar; M. Ahrens; C. Idel; B. Wollenberg; P. König; G. Hüttmann. Novel endoscope with increased depth of field for imaging human nasal tissue by microscopic optical coherence tomography. *Biomedical optics express* **2018,** *9* (2), 636–647.

[9] CTI Materials. Single Layer Graphene Oxide. https://www.ctimaterials.com/product/single-layer-graphene-oxide/ (accessed March 4, 2025).
